# A quantile regression approach to explain the relationship of Fatigue and Cortisol, Cytokine among Koreans with Hepatitis B

**DOI:** 10.1038/s41598-018-34842-5

**Published:** 2018-11-06

**Authors:** Yeonsoo Jang, Jeong Hyun Kim, Hyangkyu Lee, Kyunghwa Lee, Sang Hoon Ahn

**Affiliations:** 1 College of Nursing, Yonsei University, Seoul, 03722 South Korea; 2 Mo-Im Kim Nursing Research Institute, Seoul, 03722 South Korea; 3 Department of Internal medicine, College of Medicine, Yonsei University, Seoul, 03722 South Korea

## Abstract

Fatigue is a major symptom among patients with Hepatitis B virus (HBV). However, the physiological mechanisms regarding mediate fatigue and the relationships between fatigue, cortisol and cytokines are unclear in patients with HBV. The purpose of this study was to examine the relationships between perceived fatigue, cortisol, and cytokines in Korean patients with HBV. The mean score for overall fatigue was moderate. In linear regression analyses, TNF-α was only significant as a predictor of fatigue. In quantile regression analyses, the cortisol level was significant in the 70^th^, 80^th^, 90^th^ quantiles in the cognitive/mood fatigue subdimension, which correspond to severe levels of fatigue. IL-6 levels were significant in 90^th^ quantile in overall fatigue and in the cognitive/mood fatigue subdimension. Cortisol, IL-6, and TNF- α were related to perceived fatigue in patients with HBV, particularly in the cognitive/mood dimension. This suggests that the fatigue patterns of patients with HBV are related to their mental and mood states and physical responses, such as cortisol and cytokine levels, are correlated with the nature of the fatigue. In the clinic, interventions to manage fatigue in patients with HBV should be developed based on the characteristics of the fatigue.

## Introduction

Fatigue is the most frequently reported symptom among patients with hepatitis B virus (HBV) in clinical settings and negatively effects on their health outcomes^1–3^. Fatigue in liver disease is known as central fatigue. Central fatigue dose not correlated with traditional marker of disease activity or severity. The diagnosis of chronic liver disease includes diverse interaction such as biological, psychological and behavioral process and these can affect the clinical expression of fatigue. Fatigue in patients with liver disease has been assessed using perceived fatigue questionnaires^4^.

However, there have been few studies in HBV patients that have evaluated fatigue as it relates to physical changes in the body as can be measured by biomarkers. There has been particularly little research done relating to the interpretation of fatigue as a physical problem in patients with liver disease except for a study performed in liver transplant candidates^5^. In other chronic diseases where fatigue is the main symptom, it is recognized as a physical symptom of the patient and is explained through biomarkers. It is known that cortisol and cytokines are representative biomarkers related to fatigue. Several previous studies have attempted to identify the relationship between fatigue and various biomarkers in a variety of chronic illnesses. Previous studies have mainly evaluated IL-6 and TNF-α in cytokine analyses. TNF-α has not been shown to be related to fatigue, while IL-6 and cortisol have been reported to be positively related with fatigue^6–11^.

Meanwhile, several studies have used ordinary least squares (OLS) regression to determine the relationship between biomarkers and fatigue. However, use of the OLS method has potential limitations with respect to the interpretation and application of results. Since the OLS regression assumes a normal distribution of the data, the results provide information only on the mean of the dependent variable relative to the independent variable^12,13^. The level of fatigue in patients varies greatly in frequency and intensity, but the results of OLS only explain the data based on mean values. Therefore, the use of the OLS method to analyze fatigue has a weakness as it does not fully represent patients with high-level fatigue in whom intervention is deemed most necessary.

Quantile regression can be used as an alternative to OLS under the characteristics of these data^12^. The quantile regression method as a robust statistical methodology provides data on the relationship with outliers of predictor variables. The application of the quantile regression method to analyze fatigue is useful and suitable for interpreting the results of data with a non-normal distribution^12,14^. Therefore, the purpose of this study was to examine the relationships between fatigue, cortisol, and cytokines in patients with HBV, and compare the results using the OLS regression and quantile regression methods.

## Results

### Demographic Characteristics

The demographic characteristics of the participants in this study are presented in Table 1. The sample included a total of 143 patients (men 61%, women 39%). The mean of age was 48.9 years (SD = 11.7) and the average length of diagnosis was 15.3 years (SD = 9.5). Additionally, over 70% of the participants had taken antiviral medication. The mean levels of AST and ALT were 29.7(SD = 19.1) and 29.8(SD = 25.6) respectively. There were no significant differences in fatigue level by comorbidity (*t* = −1.826, *p* = 0.070), sex (*t* = −1.499, *p* = 0.136), taking anti-viral medication (*t* = 0.083, *p* = 0.934), and occupation (*F* = 0.977, *p* = 0.379).

**Table 1:** Demographic and Clinical Characteristics. (N = 143).

	N (%) or Mean(SD)	
Sex		
Men	86	(60)
Women	57	(40)
**Age**	48.9	(11.7)
Living with spouse		
Yes	118	(82.5)
No	25	(17.5)
Occupation		
Full-time	76	(53.1)
Part time	3	(2.1)
Not employed	64	(44.8)
HBV DNA		
<2,000 IU/ml	97	(67.8)
≥2,000 IU/ml	46	(32.2)
**Length of Diagnosis (years)**	15.3	(9.5)
Anti-viral medication		
Yes	103	(72)
No	40	(28)
**AST (IU/L)**	29.7	(19.1)
**ALT (IU/L)**	29.8	(25.6)
Comorbidity		
Yes^*^	64	(55.2)
HTN	9	(14.1)
DM	12	(18.8)
HL	5	(7.8)
GB	29	(45.3)
Others	21	(32.8)
No	79	(44.8)

^*^Multiple responses.

HTN, Hypertension; DM, Diabetes Mellitus; HL, Hyperlipidemia; GB, Gall bladder Disease; Others (G–I disease including gastric ulcer, duodenal ulcer).

### Severity of Fatigue

The mean score for fatigue in this sample was 4.3(SD 1.6), which correlates to a moderate level of fatigue. With respect to the mean scores of the four sub-dimensions, the Sensory subdimension was highest (4.58 ± 1.81) and Cognitive/Mood was lowest (3.70 ± 1.95).

### Differences of Cortisol and Cytokines by Fatigue Groups

The results for cortisol and cytokines in each fatigue group (mild-moderate-severe) are presented in Table 2. The overall mean cortisol level of participants was 10.04 ± 4.52 μg/ml (range: 2.26~22.19), that of IL-6 was 2.15 ± 1.89 pg/ml (range: 0.08~9.51) and the overall mean level of TNF-α was 1.26 ± 1.31 pg/ml (range: 0.21~13.60). The mean value for cortisol in the mild fatigue group was higher than those of the moderate and severe fatigue groups. The mean levels of IL-6 and TNF-α in the mild group were lower than those of either the moderate or severe groups. However, there were no significant differences between the mean values for cortisol and cytokines between the fatigue groups. In addition, there were no significant differences in fatigue (*t* = 0.083, *p* = 0.934), Cortisol (*t* = −1.090, *p* = 0.277), IL-6 (*t* = 1.422, *p* = 0.157), and TNF-α (*t* = 0.614, *p* = 0.540) by taking anti-viral medication.

**Table 2:** Differences in Cortisol and Cytokines by Fatigue Group. (N = 143).

	Total	Fatigue Group (Mean, SD)			Statistics^a^
		Mild (n = 66)	Moderate(n = 69)	Sever (n = 8)	*X*^2^ (*p*)
**Cortisol**	10.04 (4.52)	10.84 (4.45)	9.36 (4.55)	9.24 (4.16)	4.52 (0.104)
**IL-6**	2.15 (1.89)	1.97 (1.75)	2.30 (1.97)	2.36 (2.40)	1.79 (0.408)
**TNFα**	1.26 (1.31)	1.10 (0.79)	1.23 (0.85)	2.86 (4.36)	4.61 (0.100)

^a^Kruskal-Wallis test.

### Correlations between Cortisol, Cytokines and Fatigue

Spearman correlation coefficients were used to examine the relationships of cortisol and cytokines (IL-6, TNF-α) with fatigue (Table 3). There was a significant negative relationship between cortisol and fatigue (*r* = −0.172, *p* = 0.040). TNF-α and fatigue also showed a statistically significant positive correlation (*r* = 0.174, *p* = 0.038).

**Table 3:** Correlations of Cortisol, Cytokines and Fatigue. (N = 143).

	Cortisol	IL-6^b^	TNFα^b^
Fatigue	−0.172^*^	0.174^*^	0.268^**^

^b^Spearman’s rho test.

*p < 0.05, **p < 0.01.

### Quantile Regression and Multiple Linear Regressions for Fatigue

The results of quantile regression and multiple linear regression analyses for fatigue and the four sub-dimensions are presented in Table 4.

**Table 4:** Quantile Regression and Multiple Linear Regression Analyses for Fatigue. (N = 143).

Fatigue		Quantile Regression									OLS
		10%	20%	30%	40%	50%	60%	70%	80%	90%	
Total	C	−0.01	0.01	−0.06	−0.08	−0.09	−0.08	−0.06	−0.06	−0.07	−0.15
	I	0.18	0.21^*^	0.12	0.05	0.02	−0.04	−0.06	−0.13	−0.25^*^	0.02
	T	**0.35** ^*^	0.31	0.28	0.27	0.26	0.25	0.22	0.25	0.20	0.25*
Affective Meaning	C	−0.00	−0.01	−0.02	−0.07	−0.07	−0.04	−0.05	−0.05	−0.07	−0.08
	I	**0.13** ^*^	0.16	0.18	0.20	0.12	0.07	0.03	0.00	−0.15	0.08
	T	0.16	0.27	0.22	0.13	0.11	0.11	0.08	0.05	0.22	0.12
Behavioral/Severity	C	−0.02	−0.05	−0.04	−0.05	−0.08	−0.09	−0.10	−0.04	−0.10	−0.13
	I	**0.17** ^*^	0.14	0.06	−0.03	−0.08	−0.17	−0.26	−0.22	−0.14	−0.03
	T	0.24	0.21	**0.37** ^*^	0.36	0.32	0.32	0.68	0.37	0.69	**0.24** ^*^
Cognitive/Mood	C	0.00	−0.03	−0.03	−0.00	−0.02	−0.06	−0.13^*^	−0.13^*^	−0.17^*^	−0.15
	I	0.10	0.13	0.11	0.17	0.16	0.04	−0.10	−0.18	−0.29^*^	0.02
	T	**0.53** ^*^	**0.47** ^*^	**0.45** ^*^	**0.39** ^*^	**0.34** ^*^	0.33	0.43	**0.65** ^*^	**0.71** ^*^	**0.27** ^*^
Sensory	C	−0.06	−0.03	−0.06	−0.06	−0.07	−0.06	−0.06	−0.02	−0.05	−0.14
	I	**0.17** ^*^	0.13	0.11	0.13	0.06	−0.06	−0.10	−0.03	−0.27	0.02
	T	0.34	**0.33** ^*^	**0.29** ^*^	0.25	0.24	0.24	0.23	0.17	0.17	**0.22** ^*^

C, Cortisol; I, Interleukin-6; TNFα, Tumor Necrosis Factor Alpha.

*p < 0.05, **p < 0.01.

#### Results of Linear Regression

In the linear regression analysis, only TNF- α was determined to be a predictor. Among the sub-dimensions, TNF- α also significantly influenced the Behavioral/Severity and Sensory dimensions.

#### Results of Quantile Regression

**Figure 1:**
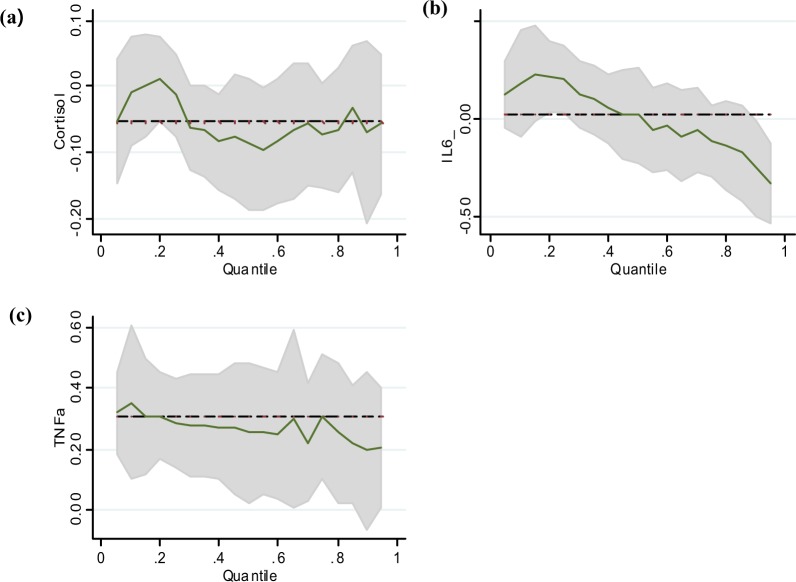
(**a**) Cortisol and Total fatigue (**b**) IL-6 and Total fatigue (**c**) TNF- α and Total fatigue in Quantile Regression.

The estimated coefficients of selected variables from the quantile regression analysis are shown as percentiles of 0.1–0.9 (Fig. 1). In the quantile regression analysis, IL-6 was a significant predictor of total fatigue at the ninetieth percentile. TNF- α was also significant in the tenth percentile.

**Figure 2:**
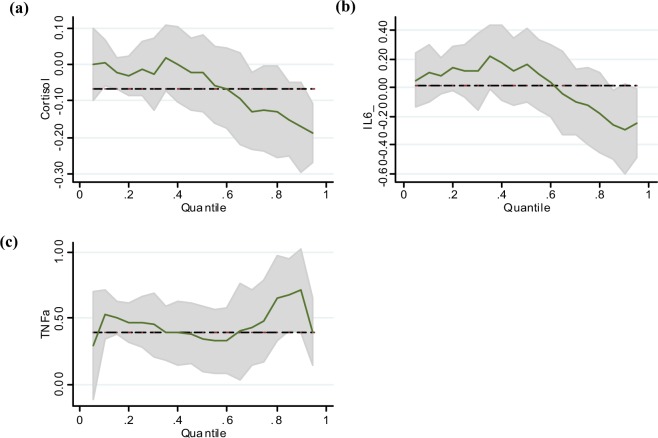
(**a**) Cortisol and Cognitive/Mood of Fatigue (**b**) IL-6 and Cognitive/Mood of Fatigue (**c**) TNF- α and Cognitive/Mood of Fatigue in Quantile Regression.

In the affective meaning and behavioral/severity dimensions, IL-6 was significant in the tenth percentile. TNF- α was significant at the 0.3 percentile in the behavioral/severity dimension. The effect of cortisol was significant at percentiles of 0.7, 0.8 and 0.9 in the cognitive/mood dimension. In addition, IL-6 was a significant predictor at the ninetieth percentile and TNF- α was significant at 0.1, 0.2, 0.3, 0.4, 0.5, 0.8 and 0.9. Lastly, in the sensory dimension, IL-6 was significant in the tenth percentile and TNF- α was significant at percentiles of 0.2 and 0.3 (Fig. 2).

## Discussion

This is the first study to examine the relationship between fatigue, cortisol, and cytokines (IL-6 and TNF- α) among Koreans with HBV using quantile regression. We conducted quantile regression to identify the relationships of cortisol, TNF- α, and IL-6 in accordance with the level of fatigue. There are two key findings of this study. First, cortisol, TNF- α, and IL-6 had significant correlations in our analysis of fatigue in Koreans with HBV. However, there were no differences in the cortisol, TNF- α, and IL-6 values between patients with different levels of fatigue in this study. Second, cortisol, TNF- α, and IL-6 were found to be predictors of fatigue in the quantile regression analysis, while TNF- α was only found to be a predictor in the linear regression analysis.

There were no differences in cortisol, TNF- α, and IL-6 between fatigue severity groups in this study. Meanwhile, decreased serum cortisol levels had a significant association with an increase in perceived fatigue. The negative correlation of fatigue and cortisol in this study is not consistent with the other studies targeting patients with severe fatigue. Previous studies have reported a positive correlation between fatigue and cortisol^6,9,10^. In other words, patients with higher levels of fatigue had higher levels of cortisol. Additionally, there were no differences of fatigue, cortisol, TNF- α, and IL-6 by taking anti-viral medication in this study. In Korea, the standard of administrating anti-viral medication is ≥2000 IU/ml of HBV-DNA in patients with HBV. Korean public medical insurance reimbursements are made according to this criteria and physicians prescribe anti-viral medication based on the standard^15^. Therefore, there might be no effect of taking anti-viral medication on fatigue because the participants may be well controlled HBV in this sample.

The characteristics of participants in this study were similar to patients with chronic fatigue syndrome who have mild to moderate levels of fatigue^16^. These symptoms, including the hypocortisolism of patients with chronic fatigue syndrome, are known to be due to chronic stress exposure and hypothalamic–pituitary–adrenal (HPA) axis dysfunction, even though the mechanism is unclear^17^. In the prior studies mentioned above, the stress experienced by the patient was relatively acute, such as stress induced by surgery, periodontitis, and less than one year of primary treatment for cancer, whereas the patients in our study had a mean duration of hepatitis 15.3 years or more. Thus, the chronicity of disease might explain the negative relationship between cortisol and fatigue seen in patients with chronic fatigue syndrome.

Another implication of the negative correlation pattern observed in chronic hepatitis B patients is that the longer the period of illness, the fewer physical symptoms may appear relative to the perceived fatigue of patient. Therefore, fatigue in HBV patients in a clinical setting seems to be interpreted more in the context of a subjective symptom than a physical symptom. The results of our quantile regression analysis support this in that cortisol was found to be a significant predictor only in the cognitive/mood dimension of fatigue. A study of the relationship between fatigue and cortisol in patients with coronary artery disease reported results similar to those in our study in that patients had lower cortisol levels as mental fatigue levels increased, unlike physical fatigue^18^. Therefore, it seems that the relationship between cortisol and fatigue might differ depending on the levels of fatigue in patients with HBV.

In addition, we compared the findings through OLS regression and quantile regression to identify the relationships between fatigue, cortisol, and cytokines in patients with HBV. We found that TNF-α was only significantly correlated with overall fatigue but cortisol and IL-6 were not correlated in our linear regression analysis even though they were significantly correlated in Pearson correlation analysis. It seems that they were not explained as predictors because the correlation coefficient values of cortisol and IL-6 were low.

This result is consistent with the fatigue studies in cirrhotic liver transplant candidates, patients with acute myeloid leukemia, and healthy active individuals^5,8,19^, but not with studies in end-stage cancer patients or rheumatoid arthritis patients^7,20^. The findings of current study using the quantile regression method may provide clues to understanding the inconsistent results from previous studies on the relationship between fatigue and biomarkers. In our analysis, TNF- α did not show a trend correlating with fatigue and was not relevant in terms of overall fatigue. Cortisol and IL-6 also displayed the same pattern. However, with respect to the sub-dimensions of fatigue, cortisol and TNF- α showed a significant association with high levels of fatigue in the cognitive/mood dimension. The finding that the reliability of biomarkers depends on fatigue is consistent with the characteristics of perceive fatigue among Korean patients with HBV^21^. In other words, cortisol and TNF- α might explain the perceive fatigue in Korean patients with HBV. These findings support the notion that fatigue of Korean patients with HBV may relate to the cognitive/mood subdimension of fatigue rather than to physical fatigue.

Previous studies have used the linear regression method to determine the relationship between fatigue, cortisol, and TNF- α. The linear regression method presents a predictive value corresponding to the mean value of the dependent variable^12,13^. This method is likely to give inadequate results in analyzing variables that have various levels, such as fatigue. Since quantile regression is a suitable method for describing values in the lower or upper percentile of a distribution^22^, the method may be helpful in identifying variables that affect patients with high levels of fatigue that are most likely within this population to require intervention.

This study has some limitations. First, there was sampling bias in this study because of the use of self-reported data and it is not generalizable. Data collection was conducted from one university medical center. Second, the results of the current study are derived from a cross-sectional design. Therefore, it is necessary to be careful in interpreting the results, and a longitudinal analysis will be needed to obtain detailed information about the relationship between fatigue, cytokines, and cortisol. Third, this study was to examine the relationship between fatigue and IL-6 and TNF- α. The results may be limited to explain the relationship of fatigue and cytokine. Further studies need to confirm the relation of fatigue with several cytokines in patients with HBV.

Despite these limitations, this study provides significant findings for patients with HBV. Our study is the first study to analyze the physical parameters of patients with hepatitis B as they relate to fatigue. Also, the use of quantile regression methods according to the level of fatigue has the methodological advantage of analyzing the full range of information as opposed to being limited to only the mean values of the data.

## Conclusion

In this study, fatigue showed a significant negative correlation with cortisol in patients with HBV; however, a significant positive correlation was seen with IL-6 and TNF-α. Cortisol, IL-6, and TNF- α were related to the levels of perceived fatigue, particularly in the cognitive/mood dimension in patients with HBV. The characteristic of fatigue in patients with chronic liver disease has been reported as a central fatigue. This type of fatigue is related to psychological and behavioral aspects of patients with chronic disease^23^. Fatigue of patients with HBV also had a tendency to affecting psychological aspects in this study. To assess perceived fatigue with other psychological factors may be important to manage fatigue as well as to assess physical expression such as biomarkers relating fatigue in this population.

In addition, TNF- α was presented as a significant predictor of fatigue in both liner regression and quantile regression analysis in this study. To decrease TNF- α might have effect to manage fatigue, but TNF-α inhibitor is known as risk factor of reactivation in HBV^24^. Although TNF-a was significantly correlated with fatigue in this study, it should be careful to use anti- TNF-α therapy in fatigue management in patients with HBV. Further studies need to be conducted to find more evidences regarding effect of anti-TNF-α to relieve fatigue in patients with HBV.

The result of this study still has limited evidence to apply fatigue management for patients with HBV. However, this suggests that the fatigue patterns of patients with HBV are related to their mental and mood states and physical responses, such as cortisol and cytokine levels, are correlated with the nature of the fatigue. In the clinic, interventions to manage fatigue should be developed based on the characteristics of the fatigue in patients with HBV.

## Methods

### Design and Setting

This study was one part of the Fatigue study project targeting Korean patients with Hepatitis B (NRF-2011-0024717). This was a descriptive correlational study to determine the relationship between cortisol, cytokines (IL-6, TNF-α) and fatigue levels in 143 patients with Hepatitis B. Data was collected between March 2012 and October 2012 from the outpatient clinic of Severance Hospital, Yonsei University Health System in Seoul, Korea.

### Sample and Sampling Criteria

This study was performed as a secondary data analysis on the Study of Fatigue related Hepatitis B reported in our previous study^21^. Participants were enrolled according to the following inclusion criteria: (1) patients aged 18 years and over, (2) patients diagnosed with HBV more than three months after the detection of HBsAg more than six months prior, (3) patients without liver cirrhosis, hepatocellular carcinoma, other infection or immune diseases, (4) patients able to give informed consent In this study, patients with interferon (IFN) therapy were not included. Over 95% of Koreans with HBV have HBV genotype C. It is known that IFN therapy is poor effect in HBV genotype C and IFN therapy is not standard therapeutic approach for Koreans with HBV^25,26^. Therefore, we excluded patients with IFN therapy in this study. The sample size was determined by G power analysis with an expected power of 0.80, significance level (a) of 0.05, and medium effect size (0.15). The sample size was calculated as 128 and ultimately 143 individuals participated in this study.

### Ethical Consideration

This study was approved by Institutional Review Board at Severance Hospital, Yonsei University Health System (IRB # 4-2011-0746) and written consent was obtained from participants. After obtaining informed consent, individuals with HBV were enrolled in this study. Participants were asked personal data and perceived fatigue. Whole blood (10 ml) was collected from participants. The experimental methods were performed in accordance with the approved guidelines.

### Measurements

#### Personal Data Questionnaire

Personal data was used to assess personal and clinical characteristics. These data consisted of self-reported demographic characteristics on sex, age, length of diagnosis, status of taking anti-viral medication for HBV, and AST and ALT level were collected along with the personal data questionnaire. This document was developed by the investigators.

#### Fatigue

Fatigue was assessed using the Revised Piper Fatigue Scale (PFS)^27^. This is a 22-numerical item self-reported questionnaire assessing fatigue experienced at the present moment. The PFS contains four dimensions of perceived fatigue: behavioral/severity, affective meaning, sensory, and cognitive. Scoring for each item ranges from zero to ten using a Likert scale. Severity of fatigue was scored as: none (0), mild (1–3), moderate (4–6), and severe (7–10) levels of fatigue. In this study, the investigators translated the questionnaire from English to Korean^28^. The reliability coefficient of the original scale was 0.97. In the present study, Cronbach α was 0.96.

#### Cortisol

Cortisol levels were analyzed using peripheral blood mononuclear cells (PMBCs) obtained from blood plasma sample drawn between 10 am and noon. From each participant, 10cc of peripheral blood was drawn for use in quantitatively measuring cortisol levels within serum using an Enzyme-Linked Immunosorbent Assay (ELISA).

#### Cytokine

Cytokine immunoassays tests were done in blood plasma sample. Detection of IL-6 and TNF-α in plasma were performed by ELISA.

### Data Analysis

Analyses were conducted using SPSS version 21 for Windows (SPSS, 2013) and Stata version 13.0 (Stata Corp, College Station, TX). Descriptive statistics were used to describe the characteristics of the samples. Spearman correlations were performed to examine the relationship between serum cytokines and perceived fatigue levels. Linear regression was used to identify predictors, which could affect fatigue level. Quantile regression analysis was used to measure the effect of cortisol and cytokines levels on the severity of fatigue. It estimates the relationship between a predictive variable and specific percentiles of the dependent variable (fatigue level).

## Electronic supplementary material

Supplementary Information

Dataset 1

## References

[1] UpToDate. *Patient education: Hepatitis B* (*Beyond the Basics*), https://www.uptodate.com/contents/hepatitis-b-beyond-the-basics (2017).

[2] Evon DM (2016). Fatigue in Patients with Chronic Hepatitis B Living in North America: Results from the Hepatitis B Research Network (HBRN). Digestive diseases and sciences.

[3] Miranda-Pettersen K (2015). The fatigue impact scale for daily use in patients with hepatitis B virus and hepatitis C virus chronic infections. Annals of hepatology.

[4] Swain MG (2006). Fatigue in liver disease: pathophysiology and clinical management. Canadian journal of gastroenterology = Journal canadien de gastroenterologie.

[5] Kalaitzakis E (2013). Hepatic encephalopathy is related to anemia and fat-free mass depletion in liver transplant candidates with cirrhosis. Scandinavian journal of gastroenterology.

[6] Bekker A (2013). The effect of intraoperative infusion of dexmedetomidine on the quality of recovery after major spinal surgery. Journal of neurosurgical anesthesiology.

[7] Evers AW (2014). Does stress affect the joints? Daily stressors, stress vulnerability, immune and HPA axis activity, and short-term disease and symptom fluctuations in rheumatoid arthritis. Annals of the rheumatic diseases.

[8] Fung FY (2013). Correlation between cytokine levels and changes in fatigue and quality of life in patients with acute myeloid leukemia. Leukemia research.

[9] Mudrika S, Muthukumar S, Suresh R (2014). Relationship between salivary levels of cortisol and dehydroepiandrosterone levels in saliva and chronic periodontitis. Journal of the International Clinical Dental Research Organization.

[10] Schrepf A (2013). Cortisol and inflammatory processes in ovarian cancer patients following primary treatment: relationships with depression, fatigue, and disability. Brain, behavior, and immunity.

[11] Torgrimson-Ojerio B (2014). Preliminary evidence of a blunted anti-inflammatory response to exhaustive exercise in fibromyalgia. Journal of neuroimmunology.

[12] Le Cook B, Manning WG (2013). Thinking beyond the mean: a practical guide for using quantile regression methods for health services research. Shanghai archives of psychiatry.

[13] Seymour J, McNamee P, Scott A, Tinelli M (2010). Shedding new light onto the ceiling and floor? A quantile regression approach to compare EQ-5D and SF-6D responses. Health economics.

[14] Yen CR (2010). An analysis of patient variables that influence intravenous patient-controlled analgesic use of morphine with quantile regression. Anesthesiology.

[15] Korean Association for the Study of the Liver. *2011_guideline_HBV*, http://www.kasl.org/bbs/index.html?code=guide&category=&gubun=&idx=&page=1&number=49&mode=view&order=&sort=&keyfield=&key= (2011).

[16] Nater UM (2008). Alterations in diurnal salivary cortisol rhythm in a population-based sample of cases with chronic fatigue syndrome. Psychosomatic medicine.

[17] Edwards LD, Heyman AH, Swidan S (2011). Hypocortisolism: An evidence-based review. Integrative Medicine.

[18] Bunevicius A (2012). Fatigue in patients with coronary artery disease: association with thyroid axis hormones and cortisol. Psychosomatic medicine.

[19] Cullen T, Thomas AW, Webb R, Hughes MG (2015). The relationship between interleukin-6 in saliva, venous and capillary plasma, at rest and in response to exercise. Cytokine.

[20] Kwak SM (2012). The relationship between interleukin-6, tumor necrosis factor-alpha, and fatigue in terminally ill cancer patients. Palliative medicine.

[21] Jang Yeonsoo, Boo Sunjoo, Yoo Hyera (2018). Hepatitis B Virus Infection. Gastroenterology Nursing.

[22] Gebregziabher M (2011). Using quantile regression to investigate racial disparities in medication non-adherence. BMC medical research methodology.

[23] Chaudhuri A, Behan PO (2004). Fatigue in neurological disorders. Lancet (London, England).

[24] Loomba R, Liang TJ (2017). Hepatitis B reactivation associated with immune suppressive and biological modifier therapies: current concepts, management strategies, and future directions. Gastroenterology.

[25] Cho I (2001). Prevalence of HBV genotypes in Korean patients with chronic hepatitis B. The Korean Journal of Hepatology.

[26] Lee SH (2001). Distribution of HBV genotypes in patients with chronic HBV infection in Korea. The Korean Journal of Hepatology.

[27] Piper BF (1998). The revised Piper Fatigue Scale: psychometric evaluation in women with breast cancer. Oncology nursing forum.

[28] Jang Y, Kim JH, Lee K (2017). Validation of the revised piper fatigue scale in Koreans with chronic hepatitis B. PloS one.

