# Author Correction: A quantile regression approach to explain the relationship of Fatigue and Cortisol, Cytokine among Koreans with Hepatitis B

**DOI:** 10.1038/s41598-020-58613-3

**Published:** 2020-01-28

**Authors:** Yeonsoo Jang, Jeong Hyun Kim, Hyangkyu Lee, Kyunghwa Lee, Sang Hoon Ahn

**Affiliations:** 1 College of Nursing, Yonsei University, Seoul, 03722 South Korea; 2 Mo-Im Kim Nursing Research Institute, Seoul, 03722 South Korea; 3 Department of Internal medicine, College of Medicine, Yonsei University, Seoul, 03722 South Korea

Correction to: *Scientific Reports* 10.1038/s41598-018-34842-5, published online 06 November 2018

This Article contains an error in Table 1 where the percentage for Comorbidity Yes and No were switched.

The correct Table 1 appears below.

**Table 1:** Demographic and Clinical Characteristics. (N = 143).

	N (%) or Mean(SD)	
**Sex**		
Men	86	(60)
Women	57	(40)
Age	48.9	(11.7)
**Living with spouse**		
Yes	118	(82.5)
No	25	(17.5)
**Occupation**		
Full-time	76	(53.1)
Part time	3	(2.1)
Not employed	64	(44.8)
**HBV DNA**		
<2,000IU/ml	97	(67.8)
≥2,000IU/ml	46	(32.2)
**Length of Diagnosis** (years)	15.3	(9.5)
**Anti-viral medication**		
Yes	103	(72)
No	40	(28)
** AST** (IU/L)	29.7	(19.1)
** ALT** (IU/L)	29.8	(25.6)
**Comorbidity**		
Yes^*^	64	(44.8)
HTN	9	(14.1)
DM	12	(18.8)
HL	5	(7.8)
GB	29	(45.3)
Others	21	(32.8)
No	79	(55.2)

^*^Multiple responses.

HTN, Hypertension; DM, Diabetes Mellitus; HL, Hyperlipidemia; GB, Gall bladder Disease; Others (G-I disease including gastric ulcer, duodenal ulcer).

