# Supplementary material for: A quantile regression approach to explain the relationship of Fatigue and Cortisol, Cytokine among Koreans with Hepatitis B
Source: Sci Rep. 2018 Nov 6;8:16434. doi: 10.1038/s41598-018-34842-5 (PMC6219556; doi:10.1038/s41598-018-34842-5)
Supplement: Supplementary file 1 — Supplementary Information [file 41598_2018_34842_MOESM1_ESM.docx]

**A quantile regression approach to explain the relationship of Fatigue and Cortisol, Cytokine among Koreans with Hepatitis B**

Yeonsoo Jang ^1, 2^, Jeong Hyun Kim ^1^, Hyangkyu Lee ^1, 2^, Kyung Wha Lee ^1^,

Sang Hoon Ahn ^3^

^1^ College of Nursing, Yonsei University, Seoul, 03722, South Korea

^2^ Mo-Im Kim Nursing Research Institute, Seoul, 03722, South Korea

^3^ Department of Internal medicine, College of Medicine, Yonsei University, Seoul,

03722, South Korea

Correspondence to Sang Hoon Ahn (E-mail: ahnsh@yuhs.ac)

**Legends of Supplement data table**

| **Label** | **Description** | **Code** |
| --- | --- | --- |
| Fatigue_feel | Duration of fatigue | 1(none) to 7(several month) |
| F_B | fatigue_behavior | 0(none) to 10(Severe) |
| F_S | Fatigue_severity | 0(none) to 10(Severe) |
| F_C | Fatigue_cognitive | 0(none) to 10(Severe) |
| F_A: | Fatigue_affective meaning | 0(none) to 10(Severe) |
| Age | Year of birth |  |
| Sex |  | 1: man, 2: woman |
| Marital Status |  | 1: married, 2: single, 3: separated,  4: bereavement, 5: divorced |
| Occupation |  | 1: full time employee, 2: part time,  3: retried, 4: student, 5: housewife,  6: others |
| Antiviral_Hx | History of antiviral medication | 1: yes, 2: No |
| Antiviral_Current | Taking antivirals currently | 1: yes, 2: No |
| AST | Serum AST |  |
| ALT | Serum ALT |  |
| Comorbidity |  | 0:No, 1: Yes |
| Cor_HTN | Comorbidity: HTN | 0:No, 1: Yes, 98: unknown |
| Cor_DM | Comorbidity: DM | 0:No, 1: Yes, 98: unknown |
| Cor_Lip | Comorbidity: Hyperlipidemia | 0:No, 1: Yes, 98: unknown |
| Cor_GB | Comorbidity: gall bladder | 0:No, 1: Yes, 98: unknown |
| LODX | Length of Diagnosis | year |
| Fatigue_Level | Level of Fatigue | 1: Low, 2: moderate, 3: high |
| Fatigue_Total | Total Fatigue score |  |
| Cortisol | Serum Cortisol value |  |
| HBV_DNA | Serum HBV DNA | 1: <2,000IU/ml, 2: ≥2,000IU/ml |
| IL6 | IL6 value |  |
| TNFa | TNFa value |  |
